# Discovery of CASP8 as a potential biomarker for high-risk prostate cancer through a high-multiplex immunoassay

**DOI:** 10.1038/s41598-021-87155-5

**Published:** 2021-04-07

**Authors:** Shiqin Liu, Fernando Garcia-Marques, Chiyuan Amy Zhang, Jordan John Lee, Rosalie Nolley, Michelle Shen, En-Chi Hsu, Merve Aslan, Kashyap Koul, Sharon J. Pitteri, James D. Brooks, Tanya Stoyanova

**Affiliations:** 1 Department of Radiology, Stanford University, Stanford, CA USA; 2 Canary Center at Stanford for Cancer Early Detection, Stanford University, Stanford, CA USA; 3 Department of Urology, Stanford University, Stanford, CA USA; 4 3155 Porter Drive, Palo Alto, CA 94304 USA

**Keywords:** Cancer screening, Prostate cancer

## Abstract

Prostate cancer remains the most common non-cutaneous malignancy among men in the United States. To discover potential serum-based biomarkers for high-risk prostate cancer, we performed a high-multiplex immunoassay utilizing patient-matched pre-operative and post-operative serum samples from ten men with high-grade and high-volume prostate cancer. Our study identified six (CASP8, MSLN, FGFBP1, ICOSLG, TIE2 and S100A4) out of 174 proteins that were significantly decreased after radical prostatectomy. High levels of CASP8 were detected in pre-operative serum samples when compared to post-operative serum samples and serum samples from patients with benign prostate hyperplasia (BPH). By immunohistochemistry, CASP8 protein was expressed at higher levels in prostate cancer tissues compared to non-cancerous and BPH tissues. Likewise, CASP8 mRNA expression was significantly upregulated in prostate cancer when compared to benign prostate tissues in four independent clinical datasets. In addition, mRNA levels of CASP8 were higher in patients with recurrent prostate cancer when compared to patients with non-recurrent prostate cancer and high expression of CASP8 was associated with worse disease-free survival and overall survival in renal cancer. Together, our results suggest that CASP8 may potentially serve as a biomarker for high-risk prostate cancer and possibly renal cancer.

## Introduction

Prostate cancer is the most common non-cutaneous cancer among men in the United States, accounting for more than one in five new cancer diagnoses. Approximately 33,000 deaths are due to prostate cancer annually, making it the second leading cause of cancer-associated deaths in men in the United States^1^. Although the 5-year survival rate for men diagnosed with localized prostate cancer is greater than 95%, it drops to 31% once the disease progresses to metastasis^1^. The high-risk prostate cancer accounts for 15% of all prostate cancer at diagnosis and commonly progresses to a lethal disease^2^. There are three common definitions of high-risk prostate cancer including pre-operative PSA levels > 20 ng/ml, Gleason score of 8–10, and clinical stage more than T2c^3^. Radical prostatectomy, surgical removal of the prostate gland, is commonly used as a treatment for high-risk prostate cancer^2,3^.

In recent years, an increasing number of cancer studies have used high-multiplex immunoassays for discovery of blood-based biomarkers^14–20^. Ideal biomarkers for prostate cancer should be specific for high-risk disease and able to be measured in a minimally or non-invasive manner. To identify new biomarkers for high-risk prostate cancer, we compared protein levels in pre-operative to post-operative serum samples from ten men with high-grade and high-volume prostate cancer by a high-multiplex immunoassay. In this study, we utilized pre-operative and post-operative serum from patients with high-risk prostate cancer (Gleason grade 4 + 5) to identify prostate cancer specific biomarkers and minimize changes in serum proteins due to patient heterogeneity including genetic background, age, pre-operative PSA levels, and other underlying medical conditions. Our study identified CASP8 as a promising protein biomarker for detection of high-risk prostate cancer and potentially renal cancer.

## Materials and methods

### Patient serum samples and tissue microarrays

De-identified patient sera utilized in this study were obtained from an existing tissue and serum repository in the Department of Urology at Stanford University. The patient-matched pre- and post-operative sera were collected from men with localized prostate cancer who either had high-risk prostate cancer (Gleason grade 4 + 5) or adverse outcomes including early recurrence and/or death from prostate cancer (Table 1). The pre-operative sera were collected from men immediately prior to surgery for prostate cancer. The patient-matched post-operative serum samples were taken between 13 days and 3 months after radical prostatectomy (Table 1). Additional serum samples were analyzed from patients with elevated serum PSA levels who underwent simple prostatectomy for BPH and were histologically confirmed to have no cancer. Tissue microarrays (TMAs) were built as previously described utilizing tissue samples from the existing tissue and serum repository in the Department of Urology at Stanford University^21–23^. Informed consent was obtained from each prostate cancer patient. All samples were collected at Stanford University under approved Institutional Review Board (IRB) protocol (IRB: 5628) and all methods were carried out in accordance with the relevant guidelines and regulations of Regional Ethics Committee and Stanford University ethical guidelines and regulations.

**Table 1:** Patient characteristics (N = 10).

Patient		Pre-op PSA	Post-op PSA	Patient characteristics			Status	Days to fail
ID #	Age			CA vol (cc)	Total high grade (4 + 5) (%)	Lymph node		
1	60	64.5	0.1	12	75	+	FAIL	19
2	67	12.2	< 0.05	3.87	80	+	FAIL	348
3	71	14.4	< 0.05	18	60	−	FAIL	15
4	60	32.8	< 0.05	5	60	−	FAIL	892
5	66	36.3	< 0.05	4.47	80	−	FAIL	13
6	62	10	< 0.05	1.57	70	−	CURE	NA
7	66	19.6	0.05	3	60	−	FAIL	945
8	67	10.84	< 0.05	5	95	−	CURE	NA
9	71	5.23	< 0.05	7	90	−	FAIL	283
10	71	29.1	< 0.05	7	80	−	CURE	NA

### Olink proteomics

**Figure 1:**
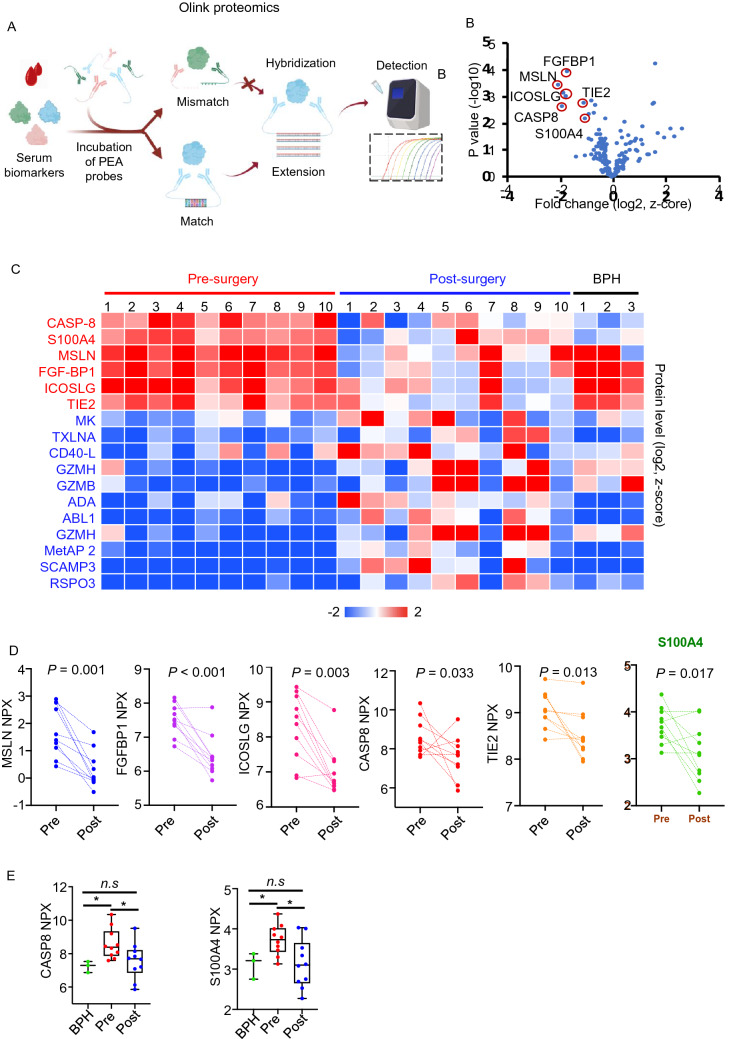
Discovery of potential serum-based biomarkers for high-risk prostate cancer though Olink proteomics. (**A**) Overview of the Olink PEA technology. Antibody pairs labelled with DNA oligonucleotides bind target protein in serum samples. Paired oligonucleotides that are brought into proximity hybridize and are amplified by a DNA polymerase. The newly created piece of DNA barcode is amplified by PCR and quantified by microfluidic qPCR. The schematic representation was generated via BioRender (https://biorender.com). (**B**) A volcano plot of proteins levels comparing pre-operative and post-operative serum samples analyzed by Olink Multiplex Immuno-oncology and Oncology II panels. (**C**) A heatmap of six proteins found to be significantly decreased and eleven proteins found to be significantly elevated after radical prostatectomy. (**D**) Normalized protein levels of MSLN, FGFBP1, ICOSLG, CASP8, TIE2 and S100A4 in comparison of preoperative and postoperative serum of prostate cancer patients. For all panels, *p < 0.05 and n.s. = not significant. Significance was determined by Student’s t-test (two-tailed). (**E**). Normalized protein levels for CASP8 and S100A4 in BPH, pre-operative and post-operative serum samples were plotted.

To discover proteins with altered levels in high-risk prostate cancer, protein levels were measured in pre-operative and post-operative serum samples from ten men with high-grade and high-volume prostate cancer (Table 1) by Olink Proximity Extension Assay (PEA) technology (Olink Bioscience, Sweden). One hundred and eighty-four human proteins associated with immuno-oncology and oncology were evaluated (Table 2). The detection of proteins was achieved by antibody-DNA oligonucleotide pairs in which DNA is hybridized upon proximity binding of the antibodies to the target, leading to formation of PCR template sequences. A real-time qPCR was utilized to quantify the DNA sequences (Fig. 1A).

**Table 2:** Olink protein panels.

Immuno-oncology panel			Oncology panel II		
ADA	CASP-8	IL-18	5′-NT	ADAM8	IFN-γ-R1
ADGRG1	FASL	IL-2	ADAM-TS 15	ESM-1	IL6
ANGPT1	FGF2	IL-33	TXLNA	EPHA2	HK11
TIE2	CX3CL1	IL-4	AREG	FASL	KLK13
ANGPT2	GAL-1	IL5	ANXA1	FADD	HK14
ARG1	GAL-9	IL6	CD207	FCRLB	HK8
CCL17	GZMA	IL-7	CXCL13	FGF-BP1	LYPD3
CCL19	GZMB	IL-8	CAIX	FR-ALPHA	MIA
CCL20	GZMH	KIR3DL1	CPE	FR-GAMMA	MSLN
CCL23	HO-1	LAP TGFB1	CEA	FUR	METAP2
CCL3	HGF	LAG3	CEACAM1	GAL-1	MIC-A/B
CCL4	ICOSLG	LAMP3	CTSV	GPC1	MK
CXCL1	IFN-GAMMA	CSF-1	CD160	GZMB	MAD H5
CXCL10	IL-1 ALPHA	MMP-12	CD27	GZMH	MUC-16
CXCL11	IL10	MMP-7	CD48	HGF	PVRL4
CXCL13	IL-12	MIC-A/B	CD70	ICOSLG	PPY
CXCL5	IL12RB1	MCP-1	CRNN	IGF1R	PODXL
CXCL9	IL-13	MCP-2	CDKN1A	ITGAV	EGF
CAIX	IL15	PTN	DLL1	ITGB5	SCAMP3
MCP-3	CD40-L	EGF	CYR61	TLR3	SEZ6L
MCP-4	CD28	PD-L1	S100A11	TGF-ALPHA	
MUC-16	TRAIL	PD-L2	S100A4	GPNMB	SPARC
NCR1	TWEAK	PDCD1	RET	TNFSF13	SCF
CD244	TNF	CXCL12	RSPO3	TNFRSF19	SYND1
KLRD1	TNFSF14	CD4	HER2	TNFRSF4	TCL1A
NOS3	TNFRSF12A	CD5	HER3	TNFRSF6B	LY9
PGF	TNFRSF21	CD8A	HER4	ABL1	TGFR-2
PDGF-B	TNFRSF4	CRTAM	WFDC2	LYN	TFPI-2
CD40	TNFRSF9	DCN	WIF-1	VEGF-A	TRAIL
CD70	VEGF-A	CD27	WISP-1	VEGFR-2	CXCL17
CD83	VEGFR-2		XPNPEP2	VEGFR-3	VIM

### Analysis of mRNA levels in clinical datasets

To evaluate the expression of genes in prostate cancer, CASP8 and S100A4 gene expression levels in normal prostate and prostate carcinoma in Oncomine and Gene Expression Omnibus were analyzed^24–28^. The log2 median centered intensity for each gene was determined for each group. To test whether the expression of CASP8 correlated with progression in prostate cancer, the expression of CASP8 was tested in existing datasets for correlation with recurrence^22^. To evaluate the expression of CASP8 in renal cancer, bladder cancer, and adrenal carcinoma, CASP8 gene expression levels in normal and cancer tissues from Oncomine and Gene Expression Omnibus were analyzed^29–36^. Student’s t-test was used to compare each two groups (normal vs prostate cancer, normal vs metastatic prostate cancer, primary prostate cancer vs metastatic prostate cancer, normal vs renal cancer, normal vs bladder cancer, normal vs adrenal carcinoma, and non-recurrent vs recurrent cancer). To evaluate whether CASP8 correlates with outcome in renal cancer, median CASP8 mRNA expression levels were correlated with overall survival in the TCGA, PanCancer, 2018 datasets. Log Rank Test was used to analyze the disease-free survival, disease specific survival, and overall survival. Survival analysis was performed in Prism 9.0 software.

### Immunohistochemistry

TMAs sections were freshly cut and heated at 64 °C for 1 h before rehydration. The slides were rehydrated with descending gradient alcohol for 5 min in each, and then incubated with 10 mM citrate buffer (pH = 6.0) at 95 °C for 30 min for antigen retrieval. Hydrogen peroxide (3%) was added to the TMA sections for 5 min. Slides were blocked with 2.5% goat serum at room temperature for 1 h. CASP8 antibody (sc-56070, 1:100, Santa Cruz) was added to the slides and incubated at 4 °C overnight. Slides were incubated with goat anti-mouse IgG ImmPRESS (TM) Secondary Antibody (MP-7452-15, Vector Laboratories) at room temperature for 1 h followed by staining with DAB kit in accord with manufacturer’s instructions (Dako). After staining with hematoxylin, slides were dehydrated and mounted. Tissue cores were scored from 0 to 2 based on staining intensity (0 is negative, 1 is low, 2 is medium/high). For each antibody, appropriate positive and negative controls were included. The TMA slides were scanned using a NanoZoomer (Hamamatsu).

### Statistical analysis

Student’s t-test was used to compare two groups and calculate p-values. The statistical significance of the differences between normal versus BPH, normal versus prostate cancer, and BPH versus prostate cancer were calculated through the normal distribution N (0,1) of z-scores. All tests were two-sided, and p-values of 0.05 or less were considered statistically significant. **p < 0.01, *p < 0.05, and n.s = not significant.

## Results

### Discovery of potential serum biomarkers for high-risk prostate cancer

We performed a high-multiplex immunoassay measurement on patient-matched pre-operative and post-operative serum samples from ten patients who had high-risk (Gleason grade 4 + 5) prostate cancer confirmed histologically at radical prostatectomy (Table 1). Seven of the ten men later developed biochemical recurrence, shown by a PSA > 0.05 ng/ml during follow-up after surgery (Table 1). The remaining 3 men showed no evidence of recurrence after > 7.5 years of follow-up. Levels of 92 human protein biomarkers related to immuno-oncology and 92 human protein biomarkers related to oncology were measured in the sera by Olink proteomics (Fig. 1A, Table 2). Ten proteins that were detected in less than 25% of the samples by the high-multiplex immunoassay were removed from our analysis. CASP8, S100A4, MSLN, FGFBP1, ICOSLG, and TIE2 were found significantly decreased after radical prostatectomy (Fig. 1B–E). Two proteins, CASP8 and S100A4, were not only significantly decreased in post-surgery serum samples but also exhibited low levels in serum samples from men with BPH (Fig. 1C–E). MSLN, FGFBP1, ICOSLG, and TIE2 were also elevated in sera from patients with BPH, suggesting that MSLN, FGFBP1, ICOSLG, and TIE2 were not specific for high-risk prostate cancer.

### CASP8 levels are elevated in recurrent and metastatic prostate cancer

**Figure 2:**
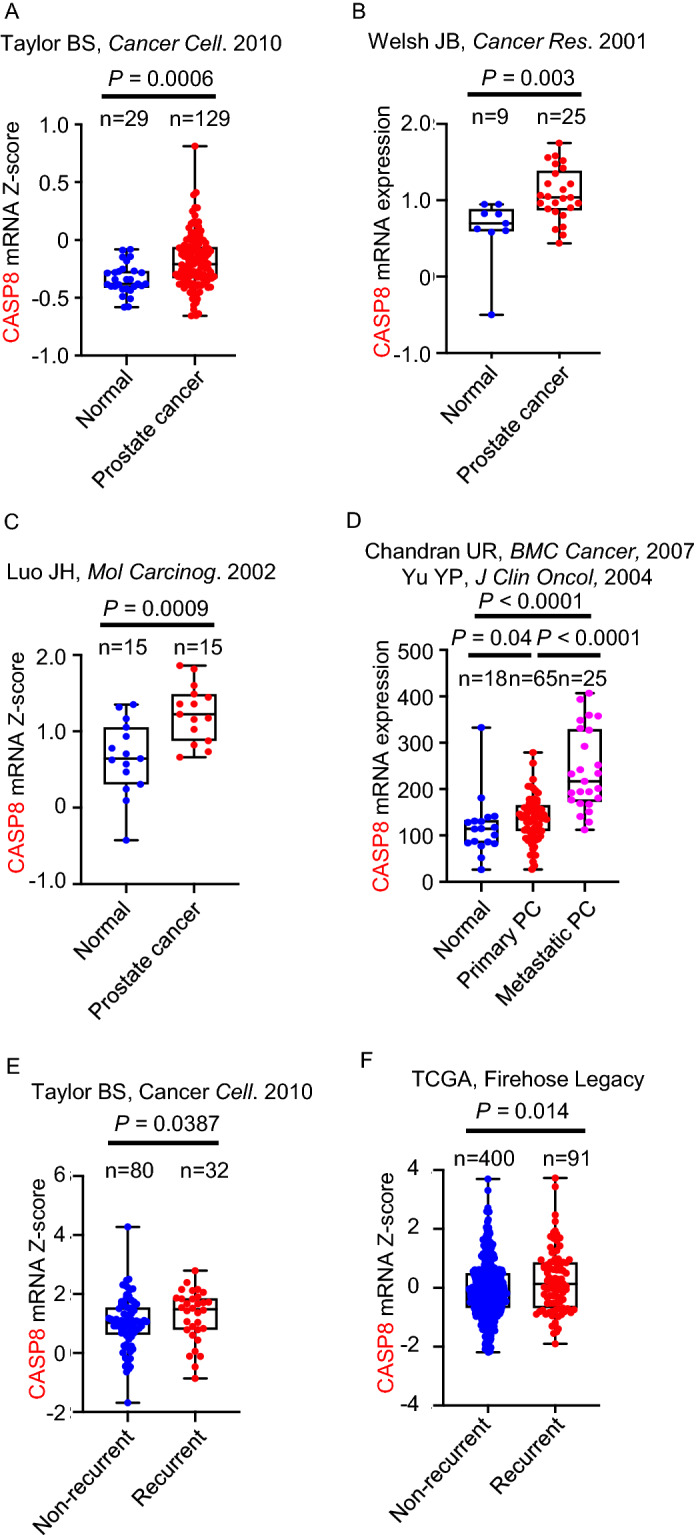
CASP8 levels are elevated in prostate cancer patients and high levels of CASP8 are associated with recurrent prostate cancer. (**A**–**E**) mRNA levels of CASP8 in clinical prostate cancer datasets. mRNA levels of CASP8 in normal prostate vs prostate cancer were from (**A**) Taylor BS, *Cancer Cell*. 2010 dataset, (**B**) Welsh JB, *Cancer Res*. 2001 dataset, and (**C**) Luo JH, *Mol Carcinog*. 2002. (**D**) mRNA levels of CASP8 were evaluated in benign/normal prostate, prostate cancer, and metastatic prostate cancer tissues utilizing Chandran UR, *BMC Cancer*, 2007 and Yu YP, *J Clin Oncol*, 2004 datasets. P values were determined by Student’s t-test (two-tailed). (**E**,**F**) mRNA levels of CASP8 in recurrent prostate cancer vs non-recurrent prostate cancer patients were plotted from (**A**) Taylor BS, *Cancer Cell*. 2010 and (**B**) TCGA, Firehose Legacy. All RNA-Seq data sets were extracted from cBioPortal and Oncomine, presented as mRNA Z-score, and Gene Expression Omnibus, presented as mRNA expression as shown.

We further analyzed the mRNA levels of CASP8 and S100A4 in four different prostate cancer datasets. CASP8 mRNA expression levels were found to be consistently elevated across datasets in primary prostate cancer when compared to non-cancerous prostate tissues while S100A4 mRNA was differentially expressed between primary cancer and non-cancerous prostate tissues only in one out of four analyzed datasets (Fig. 2A–D and Supplementary Figure S1A–D). In addition, mRNA levels of CASP8 were higher in metastatic prostate cancer when compared to primary prostate cancer (Fig. 2D). We further compared the mRNA levels of CASP8 between patients with recurrent and non-recurrent prostate cancer. CASP8 levels were significantly elevated in the cases that recurred when compared to non-recurrent cases (Fig. 2E,F). These results demonstrate that CASP8 may serve as a potential biomarker for high-risk prostate cancer, and high levels of CASP8 are associated with the risk of prostate cancer recurrence.

### CASP8 is highly expressed in prostate cancer tissues

**Figure 3:**
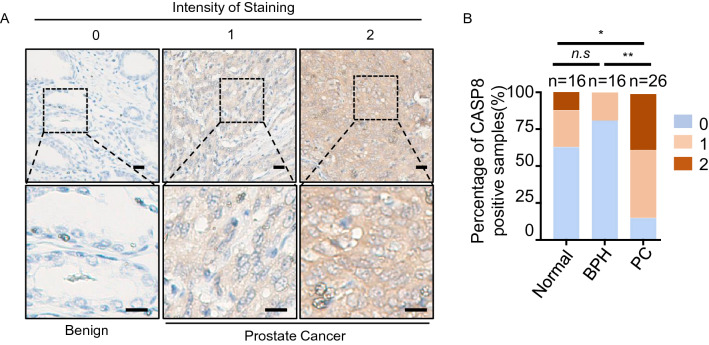
CASP8 levels are elevated in prostate cancer tissues. (**A**) IHC for CASP8 on tissue microarrays (TMAs) containing benign/normal prostate tissues (n = 16), BPH (n = 16), and prostate cancers samples (n = 26). The staining intensity of CASP8 was scored as 0 (negative), 1 (low), to 2 (medium/high). Representative images are shown. Scale bars represent 20 microns. (**B**) The protein levels of CASP8 are higher in prostate cancer when compared to normal tissues and BPH. The distribution of CASP8 intensity scores of patient samples are shown.

We further evaluated CASP8 protein levels in prostate cancer tissues. We examined the protein levels of CASP8 in prostate cancer tissue microarrays (TMAs). The TMAs contained samples of prostate cancer (N = 26), BPH (N = 16), and benign prostate tissues (N = 16) (Fig. 3A,B). The TMAs were subjected to immunohistochemical analysis for CASP8 protein expression (Fig. 3A). A higher percentage of prostate cancer samples exhibited elevated protein levels of CASP8 when compared to benign prostate and BPH tissues (Fig. 3B).

### High levels of CASP8 correlate with worse outcome in patients with renal cancer

We further tested whether CASP8 is elevated in other genitourinary cancers including renal, bladder, and adrenal carcinoma. CASP8 mRNA expression levels were elevated in renal cancer when compared to normal renal tissues across three different clinical datasets (Supplementary Figure S2A), while CASP8 mRNA expression levels had no significant differences between normal bladder tissues with bladder cancer (Supplementary Figure S2B). Moreover, CASP8 mRNA levels were not associated with recurrence in bladder cancer patients with different stages of the tumors (Supplementary Figure S2C). Additionally, mRNA levels of CASP8 were higher in adrenal carcinoma in comparison of normal adrenal tissues in one dataset (Supplementary Figure S2D).

**Figure 4:**
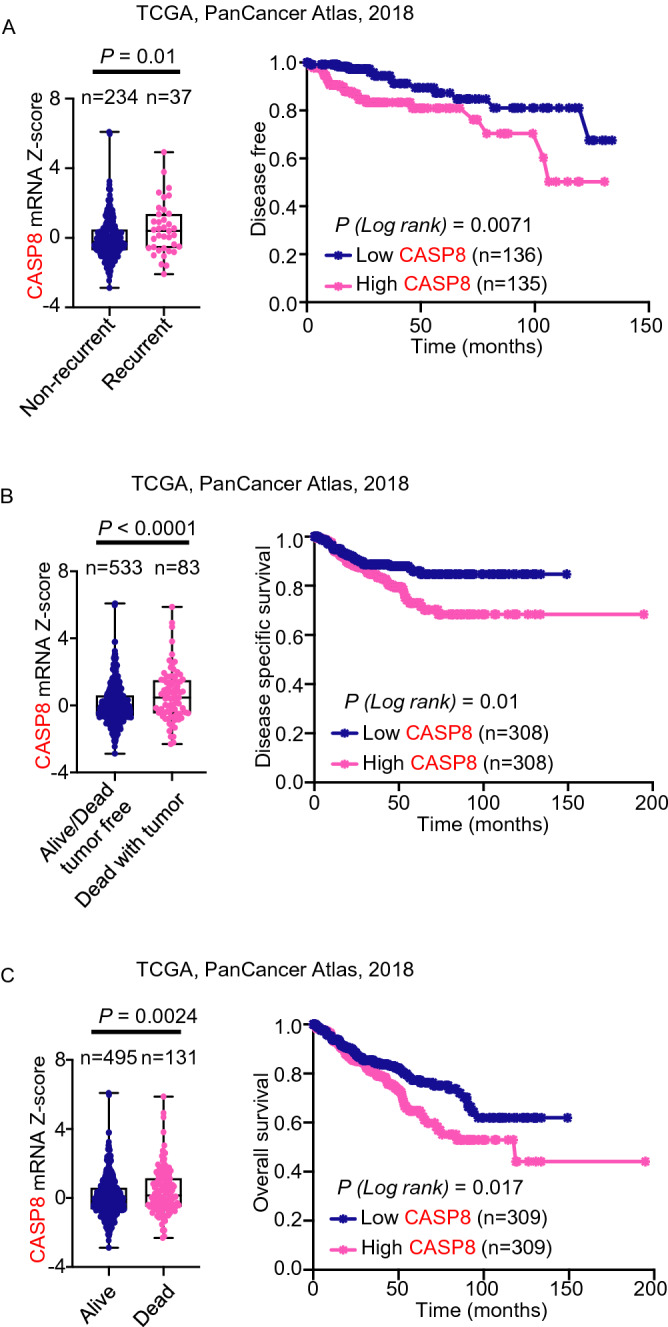
High levels of CASP8 are associated with worse outcome in renal cancer. (**A**) mRNA levels of CASP8 in recurrent renal cancer vs non-recurrent renal cancer patients were plotted (TCGA, PanCancer Atlas, 2018). Disease free survival curve was plotted. (**B**) Disease specific survival curve of patients with CASP8 high or CASP8 low expression was shown. (**C**) Overall survival of patients with CASP8 high or CASP8 low expression was presented. All RNA-Seq data sets were extracted from cBioPortal and presented as mRNA Z-score.

We next tested whether the levels of CASP8 in renal cancer were associated with more aggressive disease by analyzing the mRNA levels of CASP8 in renal cancer tissues. Patients with renal cancer recurrence exhibited higher levels of CASP8 mRNA when compared to patients who did not recur, and increased expression levels of CASP8 were associated with worse disease-free survival in renal cancer patients (Fig. 4A, p = 0.0071). Likewise, high expression of CASP8 correlated with renal cancer-specific mortality (Fig. 4B, p = 0.01) and worse overall survival (Fig. 4C, p = 0.017). Taken together, these data demonstrate that high levels of CASP8 may serve as a biomarker for unfavorable outcome in patients with renal cancer.

## Discussion

By performing a screen for proteins in pre-operative and post-operative serum from men with high-risk prostate cancer, we identified CASP8, MSLN, FGFBP1, ICOSLG, TIE2, and S100A4 proteins as candidate biomarkers for high-risk prostate cancer. Of these candidates, CASP8 and S100A4 appear to be the most attractive candidate markers since they were found at low levels in the serum from men with BPH, while several of the other candidates were also elevated in BPH. We confirmed over-expression of CASP8 mRNA and protein in prostate cancer tissues compared to controls, which is compatible with the observed drop in serum levels after prostatectomy. Intriguingly, CASP8 expression is higher in metastatic disease and recurrent prostate cancer, and is elevated in renal cancer and correlates with worse survival in renal cell carcinoma. This suggests that CASP8 could serve as a biomarker for high-risk disease, and additional validation studies are warranted.

CASP8, a caspase protein, plays a central role in apoptosis and necroptosis^37^. It has been shown that CASP8 induces cytokine production by participating in TRAIL-induced inflammatory signaling^38,39^. Inhibition of CASP8 and CASP10 associated RING protein has been demonstrated to cause a dramatic suppression of growth of different types of cancers^40^. Additionally, CASP8 D302H polymorphisms are associated with risk of several types of cancers including breast cancer, brain cancer, and prostate cancer, which indicates its potential utility for stratification of cancer patients^41–46^. It has been reported that patients with *CASP8* (− 652) gene promoter polymorphism have higher risk of progression of prostate cancer to androgen resistant state^47^. Moreover, circulating CASP8 has been associated with esophageal squamous cell carcinoma (ESCC) risk^48^.

Our screen identified several additional candidate proteins in prostate cancer samples that could have clinical implications. Mesothelin (MSLN), a tumor-associated antigen, is broadly overexpressed on various malignant tumor cells, making it as an attractive candidate for targeted therapy^49,50^. Moreover, serum MSLN has been identified as a candidate diagnostic and prognostic biomarker for pancreatic cancer^51^. Upregulation of secreted FGFBP protein may serve as a potential serum marker predicting the presence of high-risk premalignant lesions in pancreatic and colon cancer^52,53^. ICOSLG was identified as a potential biomarker of tumor progression to trastuzumab-resistant breast cancer using a pathway-based approach^54^. In addition, TIE2 was identified as the first tumor vascular response biomarker for VEGF inhibitors in metastatic colorectal cancer^55^. In patients with ovarian cancer, high circulating S100A4 transcript levels are associated with shorter progression‐free survival and overall survival, making it a potential prognostic biomarker for ovarian cancer^56^. Future work exploring the role of these proteins in prostate cancer, either as biomarkers or as potential drivers of aggressive prostate cancer, is warranted.

In summary, utilizing a high-multiplex immunoassay analysis of serum proteins from patient-matched pre-operative and post-operative serum samples from ten men with high-grade and high-volume prostate cancer, we identified CASP8 as a promising potential protein biomarker for detection high-risk prostate cancer. We further report that elevated levels of CASP8 protein and mRNA are detected in human prostate cancer tissues compared to benign and BPH patient tissues. Our study nominates CASP8 as a candidate biomarker for diagnosis of high-risk prostate cancer and warrants further large cohort validation studies.

## Supplementary Information

Supplementary Information.

## References

[1] Siegel RL, Miller KD, Jemal A (2020). Cancer statistics, 2020. CA Cancer J. Clin..

[2] Cooperberg MR, Broering JM, Carroll PR (2010). Time trends and local variation in primary treatment of localized prostate cancer. J. Clin. Oncol..

[3] Chang AJ (2014). High-risk prostate cancer-classification and therapy. Nat. Rev. Clin. Oncol..

[4] Prensner JR (2012). Beyond PSA: The next generation of prostate cancer biomarkers. Sci. Transl. Med..

[5] Force, U.S.P.S.T. (2018). Screening for prostate cancer: US preventive services task force recommendation statement. JAMA.

[6] Moradi A (2019). Beyond the biomarker role: Prostate-specific antigen (PSA) in the prostate cancer microenvironment. Cancer Metastasis Rev..

[7] Rice, M.A. and Stoyanova. T. Biomarkers for diagnosis and prognosis of prostate cancer. In: Prostatectomy. IntechOpen; (2018).

[8] Eklund M (2018). The Stockholm-3 (STHLM3) model can improve prostate cancer diagnostics in men aged 50–69 yr compared with current prostate cancer testing. Eur. Urol. Focus.

[9] Scott E (2017). Prostate cancer screening in men aged 50 to 69 years (STHLM3): A prospective population-based diagnostic study. Gronberg H, Adolfsson J, Aly M, Nordstrom T, Wiklund P, Brandberg Y, Thompson J, Wiklund F, Lindberg J, Clements M, Egevad L, Eklund M. Lancet Oncol. 2015 Dec;16(16):1667–76. [Epub 2015 Nov 10]. doi: 10.1016/S1470-2045(15)00361-7. Urol. Oncol..

[10] Loeb S, Catalona WJ (2014). The Prostate Health Index: A new test for the detection of prostate cancer. Ther. Adv. Urol..

[11] Bussemakers MJ (1999). DD3: A new prostate-specific gene, highly overexpressed in prostate cancer. Cancer Res..

[12] Tomlins SA (2016). Urine TMPRSS2:ERG plus PCA3 for individualized prostate cancer risk assessment. Eur. Urol..

[13] Stephan C (2013). Comparative assessment of urinary prostate cancer antigen 3 and TMPRSS2:ERG gene fusion with the serum [-2]proprostate-specific antigen-based prostate health index for detection of prostate cancer. Clin. Chem..

[14] Graumann J (2019). Multi-platform affinity proteomics identify proteins linked to metastasis and immune suppression in ovarian cancer plasma. Front. Oncol..

[15] Thorsen SF (2019). Gel-based proteomics of clinical samples identifies potential serological biomarkers for early detection of colorectal cancer. Int. J. Mol. Sci..

[16] Enroth S (2019). High throughput proteomics identifies a high-accuracy 11 plasma protein biomarker signature for ovarian cancer. Commun. Biol..

[17] Enroth S (2018). A two-step strategy for identification of plasma protein biomarkers for endometrial and ovarian cancer. Clin. Proteomics.

[18] Mahboob S (2015). A novel multiplexed immunoassay identifies CEA, IL-8 and prolactin as prospective markers for Dukes' stages A-D colorectal cancers. Clin. Proteomics.

[19] Soderlund S (2016). Plasma proteomics in CML patients before and after initiation of tyrosine kinase inhibitor therapy reveals induced Th1 immunity and loss of angiogenic stimuli. Leuk. Res..

[20] Shiqin, L. *et al.* Discovery of PTN as a serum-based biomarker of pro-metastatic prostate cancer. *Br J Cancer* **124**(5), 896–900 (2021). 10.1038/s41416-020-01200-0 PMC7921397 33288843

[21] Eminaga O (2016). MUC1 expression by immunohistochemistry is associated with adverse pathologic features in prostate cancer: A multi-institutional study. PLoS ONE.

[22] Hawley S (2013). A model for the design and construction of a resource for the validation of prognostic prostate cancer biomarkers: The Canary Prostate Cancer Tissue Microarray. Adv. Anat. Pathol..

[23] Howitt BE (2013). Identification and characterization of 2 testicular germ cell markers, Glut3 and CyclinA2. Appl. Immunohistochem. Mol. Morphol..

[24] Taylor BS (2010). Integrative genomic profiling of human prostate cancer. Cancer Cell.

[25] Welsh JB (2001). Analysis of gene expression identifies candidate markers and pharmacological targets in prostate cancer. Cancer Res..

[26] Luo JH (2002). Gene expression analysis of prostate cancers. Mol. Carcinog..

[27] Chandran UR (2007). Gene expression profiles of prostate cancer reveal involvement of multiple molecular pathways in the metastatic process. BMC Cancer.

[28] Yu YP (2004). Gene expression alterations in prostate cancer predicting tumor aggression and preceding development of malignancy. J. Clin. Oncol..

[29] Cutcliffe C (2005). Clear cell sarcoma of the kidney: Up-regulation of neural markers with activation of the sonic hedgehog and Akt pathways. Clin. Cancer Res..

[30] Dyrskjot L (2004). Gene expression in the urinary bladder: A common carcinoma in situ gene expression signature exists disregarding histopathological classification. Cancer Res..

[31] Giordano TJ (2009). Molecular classification and prognostication of adrenocortical tumors by transcriptome profiling. Clin. Cancer Res..

[32] Lee JS (2010). Expression signature of E2F1 and its associated genes predict superficial to invasive progression of bladder tumors. J. Clin. Oncol..

[33] Modlich O (2004). Identifying superficial, muscle-invasive, and metastasizing transitional cell carcinoma of the bladder: Use of cDNA array analysis of gene expression profiles. Clin. Cancer Res..

[34] Riester M (2012). Combination of a novel gene expression signature with a clinical nomogram improves the prediction of survival in high-risk bladder cancer. Clin. Cancer Res..

[35] Tun HW (2010). Pathway signature and cellular differentiation in clear cell renal cell carcinoma. PLoS ONE.

[36] Yusenko MV (2009). High-resolution DNA copy number and gene expression analyses distinguish chromophobe renal cell carcinomas and renal oncocytomas. BMC Cancer.

[37] Fritsch M (2019). Caspase-8 is the molecular switch for apoptosis, necroptosis and pyroptosis. Nature.

[38] Henry CM, Martin SJ (2017). Caspase-8 acts in a non-enzymatic role as a scaffold for assembly of a pro-inflammatory "FADDosome" complex upon TRAIL stimulation. Mol Cell.

[39] Sprick MR (2000). FADD/MORT1 and caspase-8 are recruited to TRAIL receptors 1 and 2 and are essential for apoptosis mediated by TRAIL receptor 2. Immunity.

[40] McDonald ER, El-Deiry WS (2004). Suppression of caspase-8- and -10-associated RING proteins results in sensitization to death ligands and inhibition of tumor cell growth. Proc. Natl. Acad. Sci. USA.

[41] Shephard ND (2009). A breast cancer risk haplotype in the caspase-8 gene. Cancer Res..

[42] Sergentanis TN, Economopoulos KP (2010). Association of two CASP8 polymorphisms with breast cancer risk: A meta-analysis. Breast Cancer Res. Treat..

[43] Zhang Y (2017). A systematic analysis of the association studies between CASP8 D302H polymorphisms and breast cancer risk. J. Genet..

[44] Cox A (2007). A common coding variant in CASP8 is associated with breast cancer risk. Nat. Genet..

[45] Lubahn J (2010). Association of CASP8 D302H polymorphism with reduced risk of aggressive prostate carcinoma. Prostate.

[46] Bethke L (2008). The common D302H variant of CASP8 is associated with risk of glioma. Cancer Epidemiol. Biomarkers Prev..

[47] Kesarwani P (2011). Influence of caspases 8 and 9 gene promoter polymorphism on prostate cancer susceptibility and early development of hormone refractory prostate cancer. BJU Int..

[48] Aversa, J. et al. *Prediagnostic circulating inflammation biomarkers and esophageal squamous cell carcinoma: A case-cohort study in Japan.* Int. J. Cancer (2019). 10.1002/ijc.32763 31671219

[49] Lv J, Li P (2019). Mesothelin as a biomarker for targeted therapy. Biomark. Res..

[50] Yakushiji H (2019). Novel single-chain variant of antibody against mesothelin established by phage library. Cancer Sci..

[51] Kendrick ZW (2014). Serum IGFBP2 and MSLN as diagnostic and prognostic biomarkers for pancreatic cancer. HPB.

[52] Tassi E, Wellstein A (2006). The angiogenic switch molecule, secreted FGF-binding protein, an indicator of early stages of pancreatic and colorectal adenocarcinoma. Semin. Oncol..

[53] Tassi E, Wellstein A (2006). Tumor angiogenesis: Initiation and targeting - therapeutic targeting of an FGF-binding protein, an angiogenic switch molecule, and indicator of early stages of gastrointestinal adenocarcinomas. Cancer Res. Treat..

[54] Nam S (2015). A pathway-based approach for identifying biomarkers of tumor progression to trastuzumab-resistant breast cancer. Cancer Lett..

[55] Jayson GC (2018). Plasma Tie2 is a tumor vascular response biomarker for VEGF inhibitors in metastatic colorectal cancer. Nat. Commun..

[56] Link T (2019). Clinical relevance of circulating MACC1 and S100A4 transcripts for ovarian cancer. Mol. Oncol..

