# Supplementary material for: Discovery of CASP8 as a potential biomarker for high-risk prostate cancer through a high-multiplex immunoassay
Source: Sci Rep. 2021 Apr 7;11:7612. doi: 10.1038/s41598-021-87155-5 (PMC8027881; doi:10.1038/s41598-021-87155-5)
Supplement: Supplementary file 1 — Supplementary Information. [file 41598_2021_87155_MOESM1_ESM.pdf]

Supplementary Information For

Discovery of CASP8 as a potential biomarker for high-risk prostate cancer through a high-multiplex immunoassay

Shiqin Liu[1,2], Fernando Garcia-Marques[1,2], Chiyuan Amy Zhang[3], Jordan John Lee[1,2], Rosalie Nolley[3], Michelle Shen[1,2], En-Chi Hsu[1,2], Merve Aslan[1,2], Kashyap Koul[1,2], Sharon J. Pitteri[1,2], James D. Brooks[2,3], and Tanya Stoyanova[1,2]*

[1]Department of Radiology, Stanford University, Stanford, CA, USA. [2]Canary Center at Stanford for Cancer Early Detection, Stanford University, Stanford, CA, USA. [3]Department of Urology, Stanford University, Stanford, CA, USA.

Supplementary Figure S1

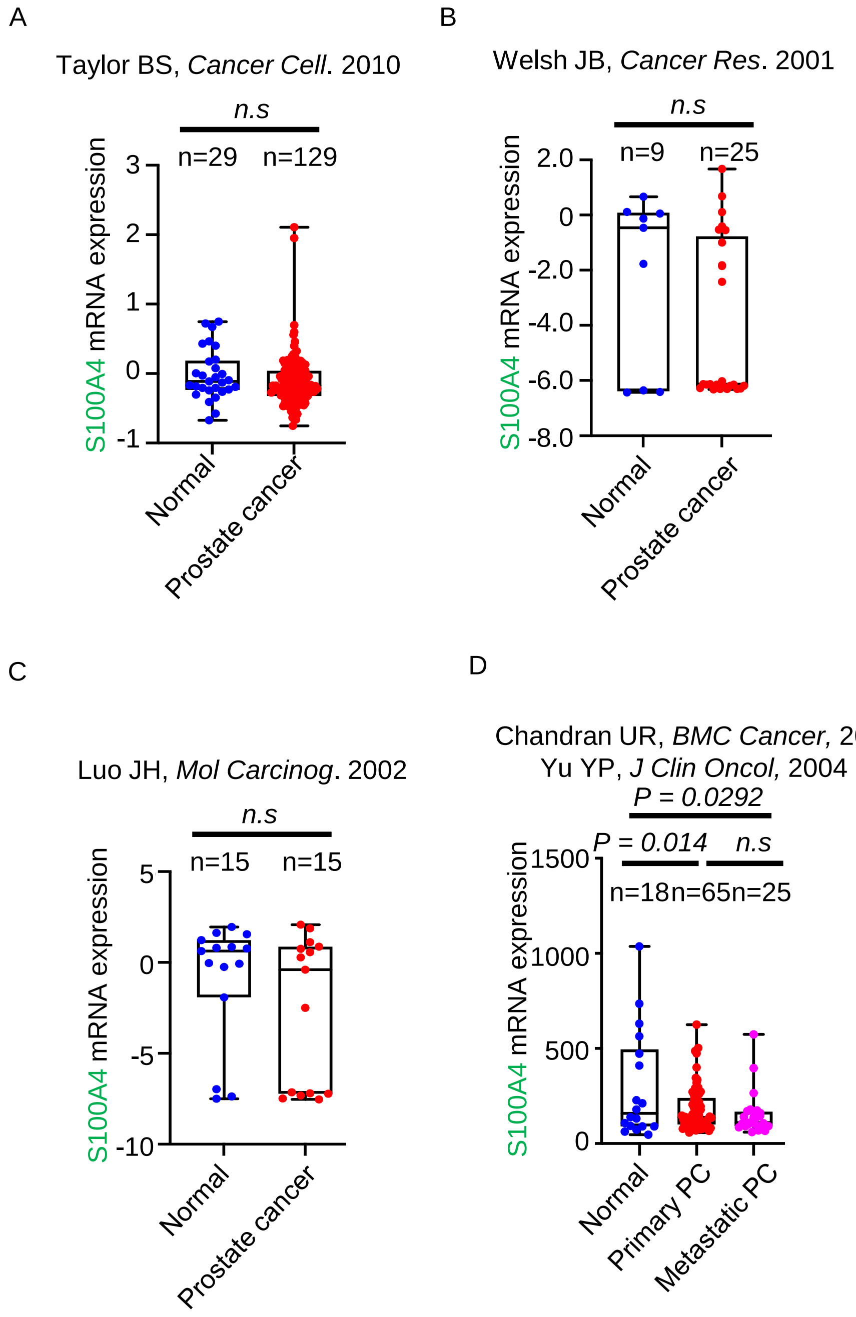

Supplementary Figure S1. mRNA levels of S100A4 are not elevated in prostate cancer. (A-D) mRNA levels of S100A4 in clinical prostate cancer datasets. mRNA levels of S100A4 in benign/normal prostate vs prostate cancer were analyzed from (A) Taylor BS, *Cancer Cell*. 2010 dataset, (B) Welsh JB, *Cancer Res*. 2001 dataset, and (C) Luo JH, *Mol Carcinog*. 2002. (D) mRNA levels of S100A4 were evaluated in normal prostate, prostate cancer and metastatic prostate cancer tissues utilizing Chandran UR, *BMC Cancer*, 2007 and Yu YP, *J Clin Oncol*, 2004 datasets. P values were determined by Student’s t-test (two-tailed).

Supplementary Figure S2

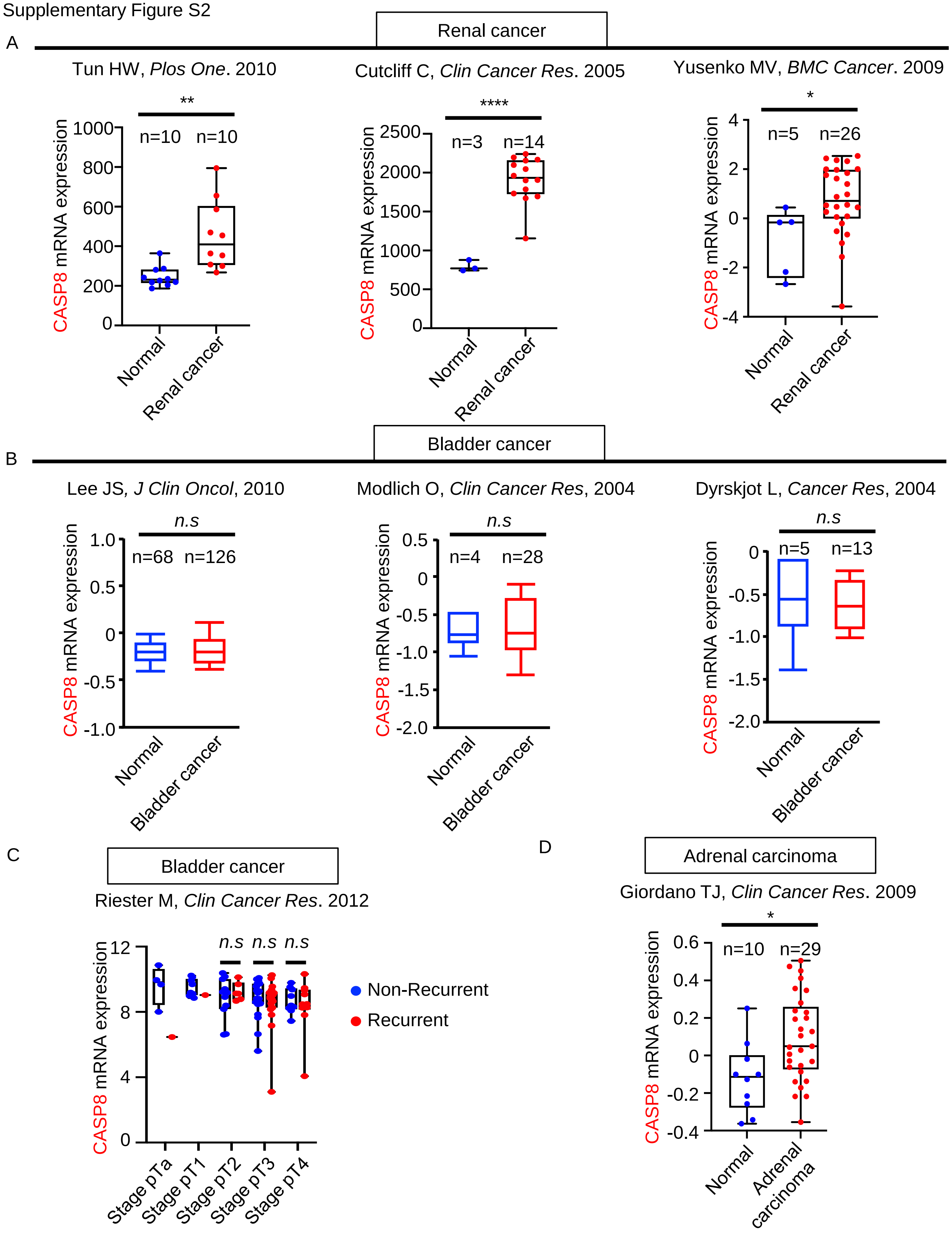

Supplementary Figure S2. mRNA levels of CASP8 in renal cancer, bladder cancer, and adrenal carcinoma. (A) mRNA levels of CASP8 in clinical renal cancer datasets. mRNA levels of CASP8 in normal renal cortex versus clear cell renal cell carcinoma were analyzed from Tun HW, Plos One. 2010, Cutcliff C, Clin Cancer Res. 2005, and Yusenko MV, BMC Cancer. 2009. (B) mRNA levels of CASP8 in clinical bladder cancer datasets. mRNA levels of CASP8 in normal bladder vs infiltrating bladder urothelial carcinoma were analyzed from Lee JS, J Clin Oncol, 2010, Modlich O, Clin Cancer Res, 2004, and Dyrskjot L, Cancer Res, 2004. (C) mRNA levels of CASP8 in non-recurrent vs recurrent bladder cancer were analyzed from Riester M, Clin Cancer Res. 2012. (D) mRNA levels of CASP8 in adrenal cortex vs adrenal cortex adenoma were analyzed from Giordano TJ, Clin Cancer Res. 2009. P values were determined by Student's t-test (two-tailed).
